# Variability of Sequence Surrounding the *Xist* Gene in Rodents Suggests Taxon-Specific Regulation of X Chromosome Inactivation

**DOI:** 10.1371/journal.pone.0022771

**Published:** 2011-08-03

**Authors:** Alexander I. Shevchenko, Anastasia A. Malakhova, Eugeny A. Elisaphenko, Nina A. Mazurok, Tatyana B. Nesterova, Neil Brockdorff, Suren M. Zakian

**Affiliations:** 1 Institute of Cytology and Genetics, Russian Academy of Sciences, Siberian Branch, Novosibirsk, Russian Federation; 2 Institute of Chemical Biology and Fundamental Medicine, Russian Academy of Sciences, Siberian Branch, Novosibirsk, Russian Federation; 3 Department of Biochemistry, University of Oxford, Oxford, United Kingdom; 4 Research Center of Clinical and Experimental Medicine, Siberian Branch, Russian Academy of Medical Sciences, Novosibirsk, Russian Federation; Florida State University, United States of America

## Abstract

One of the two X chromosomes in female mammalian cells is subject to inactivation (XCI) initiated by the *Xist* gene. In this study, we examined in rodents (voles and rat) the conservation of the microsatellite region *DXPas34*, the *Tsix* gene (antisense counterpart of *Xist)*, and enhancer *Xite* that have been shown to flank *Xist* and regulate XCI in mouse. We have found that mouse regions of the *Tsix* gene major promoter and minisatellite repeat *DXPas34* are conserved among rodents. We have also shown that in voles and rat the region homologous to the mouse *Tsix* major promoter, initiates antisense to *Xist* transcription and terminates around the *Xist* gene start site as is observed with mouse *Tsix*. A conservation of *Tsix* expression pattern in voles, rat and mice suggests a crucial role of the antisense transcription in regulation of *Xist* and XIC in rodents. Most surprisingly, we have found that voles lack the regions homologous to the regulatory element *Xite,* which is instead replaced with the *Slc7a3* gene that is unassociated with the X-inactivation centre in any other eutherians studied. Furthermore, we have not identified any transcription that could have the same functions as murine *Xite* in voles. Overall, our data show that not all the functional elements surrounding *Xist* in mice are well conserved even within rodents, thereby suggesting that the regulation of XCI may be at least partially taxon-specific.

## Introduction

X chromosome inactivation (XCI) is a developmentally regulated process, which results in heterochromatization and transcriptional silencing of one of the two X chromosomes in eutherian females [1]. Imprinted XCI occurs on the paternal X chromosome (Xp) in the preimplantation embryo of some eutherians (for example, rodents) and is further maintained in the placenta [2], [3]. Random XCI takes place on either the maternal or paternal X chromosomes after Xp is reactivated in the cells giving rise to the embryo proper.

A complex X-linked locus termed the X-inactivation centre (Xic) governs both imprinted and random XCI (reviewed in [4]). It has been shown that the initiation of XCI and propagation of silencing are mainly provided by the *Xist* gene which produces a 17 kb nuclear RNA associated with the inactive X chromosome [5]–[8]. This is the only functional element of the Xic that has been identified in all eutherians studied [9]–[11]. The studies of the Xic in mice have detected multiple elements in surrounding *Xist* with roles at different stages of XCI. Two non-coding nuclear RNA genes *Enox (Jpx)* and *Ftx* are localized 5′ to *Xist* [12], [13]. Both genes are positive regulators of *Xist* [14], [15]. The microsatellite region *DXPas34*, the *Tsix* gene (the antisense counterpart to *Xist*), regulatory element *Xite*, and а 37 kb bipartite counting element have been mapped 3′ to *Xist* (reviewed in [16], [17]). As is demonstrated, these elements in mice regulate *Xist* expression during imprinted and random XCIs and are involved in the mechanisms underlying the counting of X chromosome number per diploid set of autosomes and the choice of the X chromosome to be inactivated during random XCI. However, these regulatory elements have not been definitely identified in other eutherians.

In this study, we intended to find any conserved elements surrounding *Xist* in rodents (mouse, rat, and common voles). Mouse and rat represent Muridae rodents. These two species diverged from a common ancestor 2 million years ago [18], [19]. Voles are Arvicolidae rodents, which diverged from Muridae lineage 15–25 million years ago. We have earlier identified and described the nucleotide sequences of *Xist* in four common vole species [20]. In this work, we have extended our analysis of vole Xic further downstream of *Xist* and compared the region 3′ to *Xist* in vole, mouse, and rat. We have also determined all transcription upstream, downstream, and across *Xist* in vole, as such transcription has been suggested to regulate *Xist* expression and be involved in counting and choice function of Xic [21].

We have found that the vole sequences downstream of *Xist* have homology to the minisatellite *DXPas34* region and the *Tsix* major promoter of mouse Xic. We have demonstrated that this putative *Tsix* promoter identified in voles is a site of origin for transcription antisense to *Xist*, displaying a very similar expression pattern to mouse *Tsix*. Conservation of the *Tsix* major promoter sequences and the pattern of *Tsix* expression between vole, mouse and rat suggest a crucial role for antisense transcription in the regulation of *Xist* expression and XIC in rodents. However, we have found that the region containing the *Tsix* minor promoter and the regulatory element *Xite* in mice is replaced with the *Slc7a3* gene and its surrounding sequence in voles. This allows us to suggest that both the nucleotide sequences of these elements and the transcription associated with them are not absolutely necessary for XCI even in rodents.

## Results

### Comparative Study of the Sequences 3′ to *Xist* in Voles, Mouse and Rat

**Figure 1:**
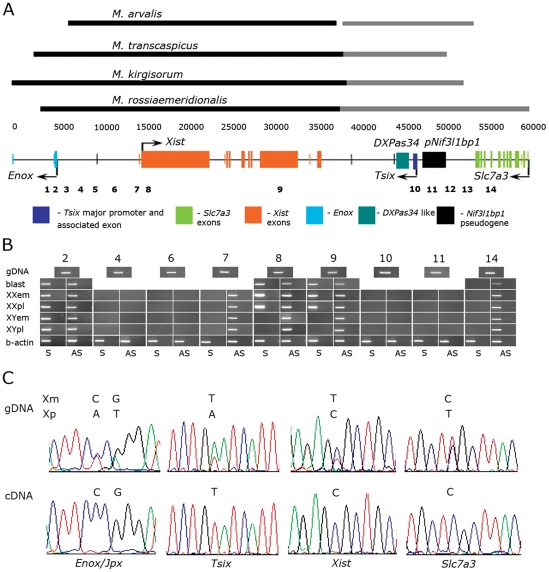
Genes, Regulatory Elements and Transcriptional Activity in Vole Xic. (*A*) Schematic representation of genes and regulatory elements detected in vole Xic by comparative sequence analysis. Lines above the scheme represent the nucleotide sequences determined for each vole species. Black lines show the sequences determined previously [20], gray lines, the sequences obtained from this study. The amplicons analyzed by RT–PCR (1–14) are denoted. (*B*) Strand-specific RT–PCR across *Xist* and its adjacent regions in preimplantation blastocysts (blast), 12,5 dpc placentas (pl) and embryos (em) of *M. rossiaemeridionalis*. Numbers above correspond to the amplicons shown on the scheme (*A*). (AS) Antisense transcript; (S) sense transcript; (gDNA) PCR of genomic DNA; (b-actin) examples of positive control of strand-specific cDNA syntheses with beta-actin primers. (*C*) Allele-specific profile of the vole *Enox/Jpx, Xist, Tsix* and *Slc7a3* gene expression in placenta of interspecific vole hybrids. Xp, X chromosome inherited from father (inactive X in placenta); Xm, X chromosome inherited from mother (active X in placenta).

We have earlier identified and described the nucleotide sequences of *Xist* in four common vole species [20]. In this study, we have extended our analysis further downstream of *Xist*, as it is known that this region in mouse comprises the *DXPas34*, *Tsix,* and *Xite* regulatory elements, which act at different steps of XCI (reviewed in [16], [17]). Several clones were isolated from phage genomic libraries for each vole species, and the sequence contig extending over 24 kb downstream of the vole *Xist* built (Fig. 1). Comparative sequence analysis shows that sequences 3′ to *Xist* of the four vole species display an overall similarity.

Then we compared the mouse and rat Xic sequences 3′ to *Xist* up to the *Tsx* gene from the UCSC Genome Bioinformatics database and found that this region of the two rodent species displayed a high similarity. In the rat sequence, we have identified all the regulatory elements mapped in the mouse Xic region (Fig. S2).

Comparison of the vole sequence 3′ to *Xist* with the corresponding mouse and rat regions detected homology to the mouse minisatellite *DXPas34* region, the major promoter of mouse *Tsix*, and some sequences surrounding these elements (Fig. 1, Fig. S3). We have not found any significant homology to the mouse minor *Tsix* promoter and the associated exon or *Xite* within the vole region studied. Surprisingly, we located the vole ortholog of the mouse *Slc7a3* gene in close proximity (5 kb) to the putative vole *Tsix* major promoter (Fig. 1, Fig. S3). Then we examined in more detail all the similarities and differences found in the region 3′ to *Xist* between vole and two other rodent species.

### *DXPas34*

**Figure 2:**
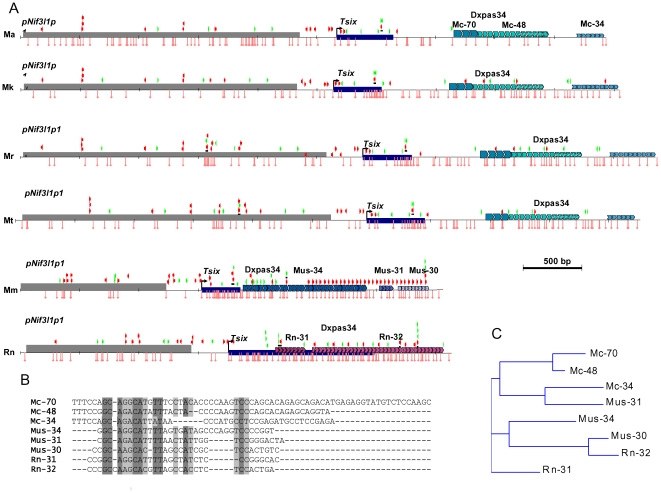
Organization of *DXPas34* Region in Voles, Mouse and Rat. Ma, *M. arvalis*; Mk, *M. kirgisorum*; Mr, *M. rossiaemeridionalis*; Mt, *M. transcaspicus*; Mm, *M. musculus*; Rn, *R. norvegicus*. Predicted CTCF-binding sites, which were found using two different consensus motifs SNMGGNGGCRGNV and GCMGCGAG indicated by red and green arrows, respectively. Lollipops at the bottom represent GpG dinucleotides. Different kinds of monomers composing *DXPas34* of vole (Mc), mouse (Mus) and rat (Rn) are shown as arrays of arrowheaded boxes. (*B*) Conserved sequence motif detected in monomers of *DXPas34* in vole, mouse and rat. (*C*) A relationship between vole (Mc), mouse (Mus) and rat (Rn) monomers of *DXPas34*.

*DXPas34* in mice has been described as a block of minisatellite repeats with a monomer of 34 bp [22]. The repeats are CpG-rich, and each monomer contains a binding site for the CTCF protein, known to be involved in XCI regulation [23]–[25]. However, our more comprehensive analysis of mouse *DXPas34* using the Tandem Repeat Finder program revealed three blocks of repeats composed of monomers of 34, 31, and 30 bp (Mus-34, Mus-31, and Mus-30) respectively (Fig. 2A). Two blocks of repeats composed of the 31- and 32-bp monomers (Rn-31 and Rn-32) have been found in rat (Fig. 2A). Three blocks of tandem repeats were detected in the four studied vole species. The first block comprises the monomers with an average length of 70 bp (Mc-70); the second, of 48 bp (Mc-48); and the third, of 34 bp (Mc-34) (Fig. 2A). The vole species differ in the copy number of monomers in the blocks. The similarity of vole monomers within blocks varies from 53 to 89 %. We managed to identify a motif that retained a high degree of conservation in all monomer types of mouse, rat, and voles (Fig. 2C). This motif may be necessary for binding of a protein factor involved in XCI regulation.

The CpG dinucleotide content and the number of binding sites for CTCF factor in the *DXPas34* region in rodents displays considerable variation (Fig. 2A). The corresponding region of rat appeared the richest in CpG content; however, it contained considerably smaller number of CTCF binding sites compared with mouse. The least CpG content and number of CTCF binding sites was observed in voles (Fig. 2A). Note that this region in *М. arvalis* completely lacked CTCF binding sites and almost lacked CpG dinucleotides. Note also that the *М. arvalis* X chromosome is predominantly active in the cells of interspecific hybrids obtained by reciprocal crosses of this species with the three remaining vole species [26].

### *Tsix* Major Promoter and Associated Exon

**Figure 3:**
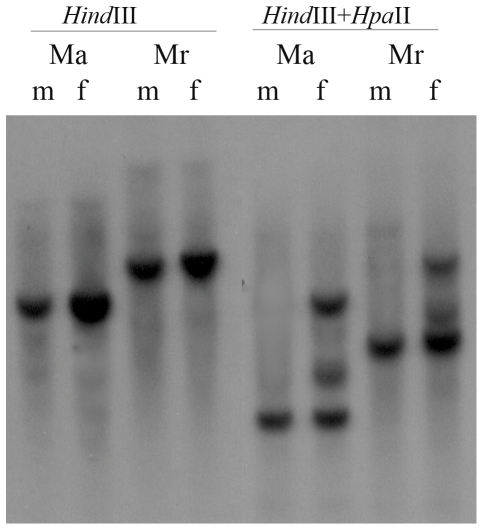
CpG Dinucleotide Methylation at 5′ Region of Vole *Tsix*. DNA methylation was detected by digestion of genomic DNA with the methyl-sensitive endonuclease *Hpa*II and subsequent blot-hybridization. Genomic DNA of adult *M. arvalis* (Ma) and *M. rossiaemeridionalis* (Mr) male (m) and female (f) was isolated from liver. DNAs were pretreated with the *Hind*III endonuclease, and then digested with *Hpa*II.

The 100-bp region located upstream of the main *Tsix* transcription start site, identified in mouse by RACE experiments [27] is the most conserved between rodent species (Fig. S3). Presumably, this region represents a basal *Tsix* gene promoter, which contains the sites necessary for initiation and regulation of its expression. The sequence immediately adjacent to the vole region similar to the *Tsix* promoter has a homology to mouse *Tsix* exon and contains a CpG island in three vole species (except *М. arvalis*). As CpG islands in this region of the mouse female inactive X chromosome are hypermethylated [28]–[30], we decided to clarify the methylation status of CpG dinucleotides in the corresponding vole region. We digested the genomic DNA isolated from *M. arvalis* and *M. rossiaemeridionalis* with the restriction endonuclease *Hpa*II, sensitive to methylation, and assayed it by Southern blot hybridization (Fig. 3). The genomic region was not methylated on the only active male X and, consequently, its DNA was completely digested with *Hpa*II. In females, carrying one active and one inactive X chromosome, in addition to the *Hpa*II-digested fraction, a fraction inaccessible for digestion with *Hpa*II due to the presence of methylated DNA on the inactive X, was also detected. Thus, at least individual CpG dinucleotides in this region of the vole female inactive X chromosome are hypermethylated.

### Pseudo *NIF3L1BP1*

Immediately adjacent to the major promoter of *Tsix*, we have found several regions homologous to the *NIF3L1BP1* gene in voles, mouse, and rat (Fig. 1, Fig. S3). They are likely to represent the remains of an ancient pseudogene the parts of which have been separated by an intensive integration of mobile elements. Pseudo *NIF3L1BP1* is the last element common to the vole, mouse, and rat regions 3′ to *Xist*. In voles, pseudo *NIF3L1BP1* is located at the boundary of the rearrangement that led to *Slc7a3* embedding.

### *Slc7a3*

Unlike mouse and rat, *Slc7a3* was found in voles at distance 5 kb from the region similar to the pseudogene *NIF3L1BP1* (Fig. 1, Fig. S3). *Slc7a3* is not located within the Xic in any other eutherians studied. In all other eutherians, the Xic region downstream of *Xist* is flanked with the protein-coding genes *Tsx, Chic1,* and *Cdx4.* In mice, *Slc7a3* is located 2.4 Mb upstream of the 5′ boundary of *Xist*. We have found that vole *Slc7a3* has the same exon-intronic structure as mouse *Slc7a3* and consists of 13 exons. The presence of the sequences homologous to promoter and the surrounding of this gene in mouse, similarity of the exon–intron structure, and absence of stop codons and frameshifts in the coding region allowed us to assume that we had identified functional *Slc7a3*, brought close to vole Xic as a result of a chromosome rearrangement.

Having discovered the rearrangement ‘;in the vole Xic region, we decided to determine the localizations of *Xist, Slc7a3, Chic1*, and *Cdx4* on the *M. rossiaemeridionalis* metaphase chromosomes. Despite *Chic1* and *Cdx4* having been obviously removed from the close proximity of *Xist* in voles, they were nevertheless located within the same cytogenetic band on the vole X chromosome as *Xist* and *Slc7a3* (Fig. S4).

### Bipartite Counting Element

The bipartite counting element is defined as a 37 kb region 3′ to *Xist* independent of *DXPas34* and *Tsix*. Deletion of the region in XY and XO embryonic stem (ES) cells results in aberrant inactivation of the only X chromosome [31]–[33], and insertion of certain sequences from the region into autosomes in XX ES cells interferes with normal counting process and blocks XCI [34]. In voles, the distance between 3′ of *Xist* and the nearest protein-coding gene *Slc7a3* is only 15 kb, of which only 4 kb displays similarity to the sequences of the mouse 37-kb bipartite counting element. This similarity is detectable across a region of about 3 kb adjacent to *DXPas34* and over 1 kb upstream of the major *Tsix* transcription start site (Fig. S3).

### Comparison of 3′ *Xist* Region in Rodents and Other Mammalian Species

**Figure 4:**
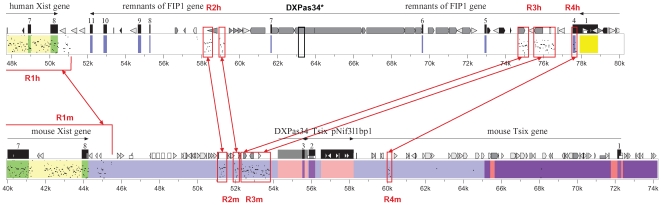
Comparison of 3′ to *Xist* Region in Mouse and Human by Reciprocal Percent Identity Plots. R1 – R4, areas of 3′ to *Xist* homology in mouse (m) and human (h) marked with red frames. Black frame shows a fragment within human transposable element which has several short dispersed regions of similarity to monomer of *DXPas34* [36]. Arrowheads and arrowheaded rectangles represent different transposable elements.

We have compared the region 3′ to *Xist* in rodents and other mammals and found no sequences homologous to *Tsix*, *DXPas34,* and *Xite* in primates (human and chimpanzee), ungulates (bovine), and carnivores (dog) (data not shown). Thus, the sequences homologous to Xic functional elements found in the mouse 3′ to *Xist* region are detectable only in the order Rodentia. However, we have identified four areas of homology between mouse and human 3′ to *Xist* regions (R1 – R4) (Fig. 4), three of which (R1-R3) have been previously reported [35]. We found that human R3 and R4 are separated by species-specific transposable elements, whereas an insertion of *DXPas34* with the 5′ region of *Tsix* occurred between mouse R3 and R4.

Previously, within a transposable element 3′ to *Xist* in human, several short dispersed regions of similarity to *DXPas34* consensus monomers were identified [36]. Nevertheless, the results of our comparative analysis show that the homology in human is not between R3 and R4 (Fig.4). Taken together these facts suggest that microsatellite repeats and adjacent sequences could have emerged from a mobile element, but that a transposon with homology to *DXPas34* found in human is probably not the genuine ancestor of the region.

### Overall Transcription Upstream, Downstream, and Across the *Xist* Gene in Voles

We analyzed the transcriptional activity at *Xist* and adjacent regions using strand-specific RT–PCR.

First, we decided to confirm the transcriptional activity of the *Slc7a3* sequence detected in voles. It has been found that this gene, as expected, is transcribed antisense to *Xist* and ubiquitously expressed in fetal tissues of both males and females (Fig. 1В, amplicon 14).

We have not identified any transcription in the intergenic region between 3′ end of *Slc7a3* and the putative transcription start site of vole *Tsix* (Fig. 1B). Thus, we have found in voles neither the sequences similar to *Xite* nor the intergenic antisense to *Xist* transcription that could have the same functions as *Xite* in mouse.

Antisense to *Xist* transcription was detected between the putative vole *Tsix* promoter and *Xist* promoter in the embryo and placenta of both males and females. Transcription in vole is identical to the transcription of the mouse *Tsix* (Fig. 1B).

*Xist* expression was revealed only in female tissues. It fits the vole *Xist* transcription unit reported previously (Fig. 1B).

Transcription neither sense nor antisense to *Xist* was detected in the intergenic region starting 2 kb upstream of the *Xist* promoter and continuing up to the GpG island near the *Enox* (*Jpx*) gene regions (Fig. 1В, amplicons 6 to 4). We did however identify transcription both sense and antisense to *Xist* transcription in the region of the vole *Enox* (*Jpx*) putative promoter. In mouse, similar sense–antisense transcription in this region occurs because of the bidirectional activity of *Enox* (*Jpx*) promoter [21].

### Allele-Specific Profile of Sense and Antisense Transcription in the Vole Xic

Differences in the Xic nucleotide sequences between the vole species allow us to determine the allele-specific profile for *Slc7a3*, *Tsix*, *Xist,* and *Enox* (*Jpx*) expressions. For this purpose, we used 12.5 dpc vole XX placentas obtained by interspecies crosses with imprinted inactivation of the paternal X chromosome.

The electrophoretic patterns of sequencing reactions for PCR products of these genes obtained from the genomic DNA of hybrid placentas displayed double peaks at the polymorphic positions, thereby confirming the presence of both parental alleles in the hybrid placentas (Fig. 1C). Sequencing of the PCR products obtained from the cDNA of hybrid XX placentas demonstrated that only *Xist* was expressed from the inactive X chromosome, whereas the remaining genes *Slc7a3*, *Tsix,* and *Enox* (*Jpx*) were expressed from the active X chromosome.

### Exon–Intronic Structure and Boundaries of the Vole *Tsix* and *Enox* Genes

To determine the exon–intron structure and transcription boundaries for *Tsix* and *Enox* in voles, we used 5′ and 3′ RACE and strand-specific RT–PCR. The RNA for 5′ and 3′ RACE was isolated from 12.5 dpc XX and XY embryos and placentas of *M. rossiaemeridionalis* and *M. arvalis.*

### *Tsix*

**Figure 5:**
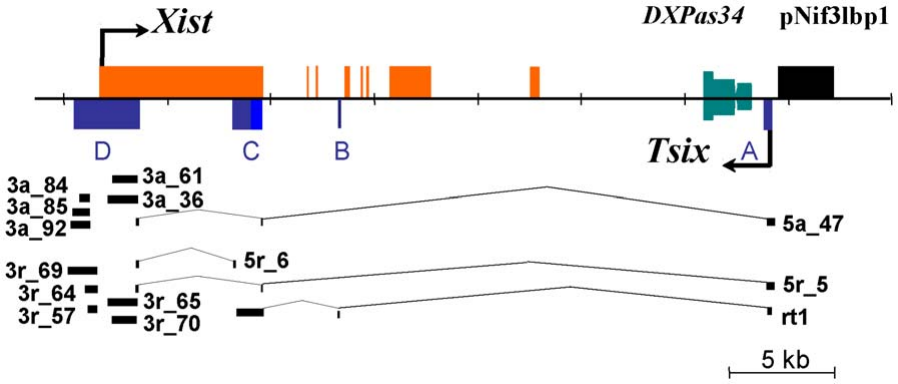
Exon-Intronic Structure of the Vole *Tsix* Gene Obtained from RACE Experiments and Strand-Specific RT-PCR. Genomic map of vole *Xist* and *Tsix* are shown; exons of *Xist* and *Tsix* are indicated by orange and indigo boxes, respectively. Clones obtained by RACE and RT-PCR are aligned under the map. Clones, 3a_n of *M. arvalis*, and 3r_n of *M. rossiaemeridionalis*, were isolated by 3′RACE. Clones, 5a_n of *M. arvalis*, and 5r_n *M. rossiaemeridionalis*, were isolated by 5′RACE. Rt1 represents a strand-specific RT-PCR product obtained from *M. rossiaemeridionalis* placenta using primers Bt11 – SNTR (Table S2).

The 3′ RACE primers for *Tsix* were designed for the regions −100 and +1500 bp relatively of *Xist* transcription start site (Table S2). The PCR products obtained by RACE were subcloned and sequenced. Analysis of the sequences of 3′ RACE products demonstrated that *Tsix* transcription in voles terminated at multiple sites encompassing the *Xist* transcription start site (Fig. 5). The most distant *Tsix* transcription termination site was detected at position –1279 bp from the *Xist* transcription start site.

The *Tsix* gene-specific primers for 5′ RACE were designed for the region +1200 bp relatively to *Xist* transcription start site. For *M. arvalis* and *M. rossiaemeridionalis,* 5′ RACE clones with a length of 600 bp were obtained (Fig. 5). The first 134 bp in the clones correspond to the sequence located immediately after the primer. The next 110 bp correspond to the sequence of *Xist* exon 1, located at a distance of 6 kb from the first homologous region. The remaining 450 bp are identical to the newly determined genomic sequence localized 12 kb downstream of *Xist* exon 8. This fragment is homologous to the mouse exon located downstream of the major *Tsix* start site. In addition, we found clones with regions of continuous 600–800-bp homology to *Xist* exon 1 detected after the 134-bp region; these homology regions started 1.5 kb downstream of the 5′ boundary of the 110-bp fragment. Presumably, these clones were truncated at the 5′ end due to a premature termination of reverse transcription. To obtain the complete structure of the *Tsix* transcript isoform, we carried out strand-specific RT-PCR using the primer set positioned within *Xist* exon 1 sequence identically to the 600–800-bp region and in the vicinity of the reported *Tsix* major transcription start site. As a result, we amplified a 2 kb cDNA fragment. Part of this fragment with a length of 1.4 kb was identical in its sequence to *Xist* exon 1, which corresponded to the 600–800-bp fragments from its 3′ end and to 110-bp fragment from its 5′ end; the next 75 bp was homologous to *Xist* intron 3; and 400 bp at the 5′ end corresponded to the sequence similar to the mouse exon located after the major *Tsix* transcription start site.

Thus, the RACE experiments allowed us to detect four exons (designated A, B, C, and D) in the vole *Tsix* gene (Fig. 5). Exon A (419 bp) is localized 12 kb downstream of the end of *Xist* exon 8. It corresponds to the exon found in mouse after the major *Tsix* start site reported in [27]. Exon B has a length of 121 bp and is localized to *Xist* intron 3. Its sequence is a fragment of B1 element. Exon C (1.4 kb) is located near the 3′ boundary of *Xist* exon 1. Several variants of alternative splicing were detected for this exon. Exon D (3.2 kb) terminates 1279 bp upstream of the *Xist* transcription start site and spans 2.9 kb of *Xist* exon 1. It corresponds to the terminal exon of mouse *Tsix* reported in [27], [37].

**Figure 6:**
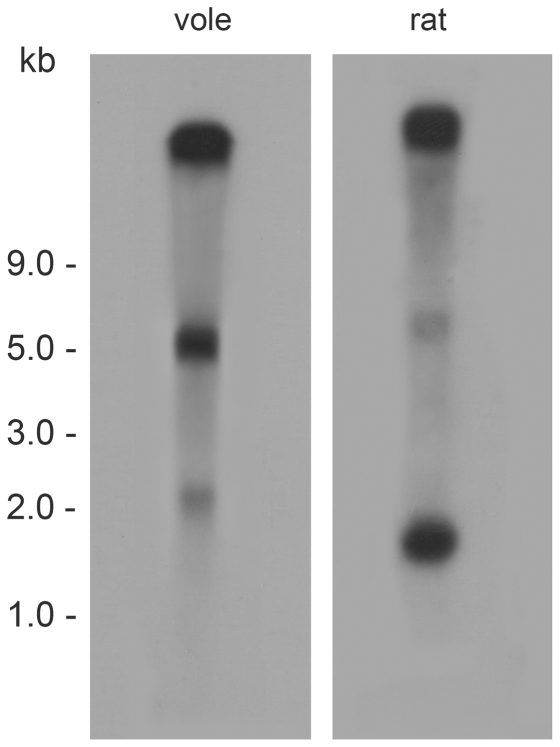
Northern Blot Analysis of *Tsix* Expression in Vole and Rat 14 dpc Male Embryos. About 5 µg of poly(A)+RNA from each species was hybridized with the species- and strand- specific probe corresponding to the exon located after the major *Tsix* start site in mouse [27].

To detect *Tsix* transcript in vole tissues, Northern blot hybridization with strand-specific probe corresponding to exon A was performed. Three major hybridization signals were observed (Fig. 6). The first high molecular band appears to represent unspliced *Tsix* transcript and to correspond in size to the whole transcription unit. The second major band is about 5 kb and presumably represents spliced transcripts containing all the four vole *Tsix* exons, total size of which is 5,14 kb. The third band detected is about 2,2 kb. In summary, the results show that the *Tsix* transcript is only partially spliced and its main portion is present in an unspliced form.

### *Enox*

We performed both 5′ and 3′ RACE to identify boundaries of *Enox (Jpx)* and its antisense counterpart. However, only 3′ RACE for *Enox (Jpx)* and 5′ RACE for its antisense transcript was successful. Gene-specific primers were designed within region 2 for 3′ RACE of *Enox (Jpx)* and within region 3 for 5′ RACE of transcript antisense to *Enox (Jpx)* (Fig. 1A, Table S2). All clones obtained in 3′ and 5′ RACE were identical to the genomic DNA sequences located immediately after the primers. The nucleotide sequences of the 5′ and 3′ RACE clones were deposited with Gene Bank (accession numbers [GenBank:JF519003], [GenBank:JF519004]). We found that vole *Enox (Jpx)* terminated 840 bp downstream from the region homologous to the mouse *Enox (Jpx)* promoter, while the antisense transcription start site was mapped within the *Enox (Jpx)* transcription unit 342 bp before its 3′ end. Thus, in vole, both *Enox (Jpx)* and its antisense counterpart represent a single exon transcription unit, which taking into account RT-PCR data (Fig. 1A,B) spans about 1500–2000 kb.

### Transcription Antisense to *Xist* in Rat

We searched for the mRNA and EST antisense to rat *Xist* at the Blat server (http://genome.ucsc.edu/cgi-bin/hgBlat). Two spliced RNAs were detected (Fig. S5). EST [GenBank:CF978550] has three exons. The first exon is within the block of tandem repeats *DXPas34* and does not coincide with any known mouse or vole *Tsix* exons. The second exon coincides with vole exon C and the third, with the terminal *Tsix* exon of vole and mouse. This RNA seems to be a spliced variant of the rat *Tsix* transcript.

The other RNA, [GenBank:AY539944], comprises ten exons and contains a native open reading frame encoding a hypothetic protein LRRGT00193. This cDNA is referred as the gene *Lrrrn* [38], [39], [40], the transcription of which has been detected in the rat liver. The exons of this gene are localized in the region homologous to the *Xite* regulatory element, in *Tsix* exon downstream of the major promoter and in *Xist* introns (Fig. S5A). Neither vole nor mouse retains the native open reading frame of the rat *Lrrrn* gene.

Short unspliced RNA immediately downstream region homologous to the *Enox (Jpx)* transcriptional start site was also identified EST [GenBank:CK839650, GenBank:BE109826].

Using strand-specific mRNA from 14 dpc rat placentas and embryos, we confirmed the exon-intronic structure of *Tsix* and the transcription corresponding to *Enox (Jpx)* identified by EST analysis (Fig. S5A). The nucleotide sequences of cDNA obtained in the experiments are deposited with the Gene Bank under accession numbers [GenBank:JF519002], [GenBank:JF519001], [GenBank:JF519000]. We also confirmed by RT-PCR that in rat *Tsix,* transcription passed through the *Xist* gene promoter. Northern blot hybridization with a strand-specific probe representing the exon which follows the major *Tsix* start site revealed in rat a high molecular weight transcript, presumably corresponding to unspliced *Tsix* and two spliced isoforms of ∼6 kb and ∼1,8 kb (Fig. 6).

## Discussion

### Variability of Transcription of *Xist* Surrounding in Rodents

In this study we identified in vole and rat 5′ to *Xist* transcription corresponding to the mouse *Enox (Jpx)* gene. We also found in vole transcription antisense to *Enox (Jpx)* which was previously described in mouse [21]. However, the transcription boundary and its start site in mouse and vole differ both for *Enox (Jpx)* and its antisense transcript. Vole *Enox (Jpx)* transcript is not spliced and the transcription unit spans about 2 kb from the initiation site, while mouse *Enox (Jpx)* is transcribed through tens of kb and gives spliced mRNA containing up to 5 exons [12], [13]. It should be noted that vole *Enox (Jpx)* transcription initiates from the species-specific CpG-rich region and obviously transcriptional regulation of the gene differs in vole and mouse. Thus, we can propose that the regulation of XCI by transcription associated with *Enox (Jpx)* is not the same in vole and mouse. It is intriguing that *Enox (Jpx)* transcription is more similar in mouse and human [12], [13] than in mouse and vole. Finally, it should be also noted that *Enox (Jpx)* is not completely silenced on the inactive X chromosome during random inactivation and is biallelically expressed in female mice [13], [15]. However, *Enox (Jpx)* appears to be expressed only on the active X chromosome in vole during imprinted inactivation. The difference in *Enox (Jpx)* expression between vole and mouse could be ascribed in equal degree both to species-specific features and different function of the gene during imprinted and random inactivation. Further studies are needed to clarify these issues.

Our results demonstrate that in both rat and vole there is expression antisense to *Xist* which corresponds to mouse *Tsix*. It starts from the region homologous to the mouse *Tsix* major promoter and ends around *Xist* transcription start site. The vole *Tsix* gene has an expression pattern similar to that of mouse *Tsix.* Both vole and rat *Tsix* transcripts cover the *Xist* promoter, which, as has been demonstrated for mouse, is obligatory for *Tsix* functioning in XCI [41]–[43]. Thus, it is most likely that similar to the mouse *Tsix,* the vole and rat *Tsix* is able to regulate *Xist* expression. However, this requires additional confirmation. Similar to mouse the vole and rat *Tsix* RNA undergoes, at least in part, alternative splicing. The terminal exons of the *Tsix* genes in mouse, rat and vole are identical, whereas the remaining exons differ between rodent species. It has been shown that many exons in the noncoding RNA genes of Xic, such as *Xist* and *Enox* (*Jpx*), originated from mobile elements of various classes [9]. In this work, we have found another example of how a part of a species-specific SINE is present in spliced mature RNA of vole *Tsix* and represents one of its exons. This example illustrates the idea that integration of mobile elements into noncoding RNA genes of Xic continues in contemporary eutherian species and also supports the assumption that the emergence of exons from mobile elements is a general way of evolution and rearrangement of genes encoding large nuclear regulatory RNAs. However, the differences in the exon–intronic structures of the rodent *Tsix* genes together with the data on the absence of the differences in the ratio of spliced and unspliced *Tsix* transcripts [37] and the absence of XCI abnormalities caused by mutations of mouse *Tsix* splicing sites [44] confirms the earlier assumption that *Tsix* splicing is not necessary for its normal function.

It should be noted that transcription in the region of the *Tsix* major promoter is bidirectional in mouse [36], however, we only identified transcription antisense to *Xist* in vole. *Xite* is present and expressed in mouse [35], but not in vole. Tissue-specific protein-coding gene *Lrrrn* is transcribed through *Xite*, *Tsix*, and *Xist* in rat [38]–[40], but is not even found in mouse and vole. Thus, comparative analysis data has shown that many transcripts that surround the *Xist* gene in rodents are taxon-specific. The taxon-specific transcripts either are able to influence XCI (as *Xite*), or are not absolutely linked with it (as *Lrrrn*).

### Conservation of the Xic 3′ to *Xist* Elements in Rodents and Other Eutherians

Comparison of the vole, mouse, and rat sequences has demonstrated that the most conserved of all the known regulatory elements downstream of *Xist* in rodents are the regions of *Tsix's* major promoter and minisatellite repeats. Their conservation and necessity in rodents suggests that they are absolutely essential for XCI in this mammalian order. Note that the region of the *Tsix* major promoter is the most conserved in rodents. However, targeted deletions of the *Tsix* promoter in mice do not impair *Tsix* function in random XCI [36].

As the sequences homologous to mouse *DXPas34* and *Tsix* major promoter are only detected in vole and rat, but not in others mammals, we could propose that this region emerged *de novo* in Rodentia, most likely as a result of the transposition and subsequent amplification of the same sequences which led to the formation of minisatellite repeat arrays. Note that transcription antisense to *Xist* is also not well conserved in eutherians. The transcript antisense to *XIST* is revealed in human, but does not overlap the *XIST* promoter [45]–[47]. Moreover, it is coexpressed with *XIST* from the inactive X chromosome in fetal and neonatal female cells and does not downregulate *XIST*, suggesting that this species-specific transcription is not linked with XCI. Thus, regulation of inactivation by *Tsix* may have arisen in the evolution quite recently and apparently is unique to rodents.

As voles lack the regions homologous to the *Tsix* minor promoter and *Xite*, we can assume that they are not absolutely necessary for XCI in eutherians. Moreover, as *Xite* sequences are only detected in mouse and rat, we cannot exclude that this element has emerged and evolved only in Muridae.

In all eutherians, for which whole genome sequences were obtained, Xic 3′ to *Xist* is flanked by the protein-coding genes *Tsx*, *Chic1* and *Cdx4* [9]. There is a speculation that others besides the known regulatory elements of XCI may lie within the region of these protein-coding genes (reviewed in [16]). The rearrangement detected in the vole Xic region demonstrates that the presence of protein-coding genes *Tsx*, *Chic1*, and *Cdx4* and the sequences linked with them downstream of the 3′ *Xist* boundary are not necessary for XCI. The Xic rearrangement has evolved and, as a result, as little as 15 kb of the sequences downstream of *Xist* characteristic of this region in mouse appear sufficient for normal imprinted and random XCI regulation in vole.

In this study we found that not all functional elements flanking *Xist* in mice were well conserved even within rodents. Non-coding RNA transcripts in the region surrounding *Xist* can appear, disappear, change their promoters, exon-intron structure and borders. No common conserved elements located 3′ to *Xist* have been identified in eutherians, suggesting that the XIC functional elements responsible for ‘counting’, ‘choice’, regulation of *Xist* and XCI may be at least partially taxon-specific.

## Methods

### Ethics statement

The study was carried out according to "The Guidelines for Manipulations with Experimental Animals." The study was approved by the Ethical Committee of the Institute of Cytology and Genetics, Novosibirsk, permit number: (order of the Presidium of the Russian Academy of Sciences of April 02, 1980 no. 12000–496).

### Screening of phage genomic libraries of common voles from the genus *Microtus*

The phage genomic libraries of a male *Microtus arvalis,* female *M. rossiaemeridionalis,* female *M. kirgisorum,* and female *M. transcaspicus* in the λ *DASH* II (Stratigene) vector [20] were screened. The amplified sequences downstream the exon 8 of *M. rossiaemeridionalis Xist* and the protein-coding regions of the mouse genes *Cdx4*, *Chic1*, *Slc16a2*, and *Slc7a3* were used as probes for screening. The manipulations with the libraries were performed according to the Stratigene recommendations. Hybridization was conducted on Colony/Plaque Screen NEN Life Science Product membranes according to the manufacturer's instructions.

### DNA sequencing and analysis

The recombinant DNA sequences from phage clones were subcloned into plasmid vectors and sequenced using both the universal and specific primers. The sequencing reactions were carried out using a Big Dye Terminator v. 3.1 kit. The reaction products were analyzed in ABI Prism automated sequencers. The DNA sequences localized 3′ to *Xist* were determined in both strands for each of the four vole species and joined with the earlier obtained sequences containing the vole genes *Enox* (*Jpx*) and *Xist* which were deposited with the GENE BANK under accession numbers [GenBank:AJ310129] (*M. аrvalis*), [GenBank:AJ310130] (*M. rossiaemeridionalis*), [GenBank:AY090554] (*M. kirgisorum*), and [GenBank:AJ310127] (*M. transcaspicus*).

Comparative sequence analysis was conducted using the following software packages: BLAST [48], http://www.ncbi.nlm.nih.gov/ ] for searching for homologous sequences; Tandem Repeat Finder 4 [49] for identifying tandem repeats; RepeatMasker [50] (http://www.repeatmasker.org) for detecting mobile elements; Fasta [51] and CLUSTALX [52] for aligning two and more sequences (the programs and data are available at http://genome.ucsc.edu/, http://www.ensembl.org/, and http://bio.cse.psu.edu/); and PipMaker [53] for conducting genomic analysis of extended loci.

The following sequences of mouse, rat, bovine, dog, chimpanzee, and human, extracted from the corresponding databases of sequenced genomes at the UCSC Genome Bioinformatics Site (http://genome.ucsc.edu/), were also used in the comparative analysis: mouse Feb 2006 (mm8) assembly range = chrX: 99509137–100404904; rat June 2003 (rn3) assembly range = chrX:91358074–91899712; dog May 2005 (canFam2) assembly canFam1_dna range = chrX:60100000–60735000; chimpanzee Mar 2006 (panTro2) assembly range = chrX:75160560–75392648; human Mar. 2006 (hg18) assembly range = chrX:72449111–74160153.

### RNA and DNA isolation, reverse transcription, and PCR

RNA and DNA were isolated using a Tri Reagent (Sigma) kit from pooled preimplantation vole blastocysts, 12.5 dpc vole embryos and placentas. The cDNA was synthesized using total RNA by SuperScript III (GIBCO-BRL) reverse transcriptase at 50–55°C according to the manufacturer's instructions.

The strand-specific primers used for reverse transcription and cDNA amplification are listed in Table S1. Each reverse transcription reaction mixture was additionally supplemented with the strand-specific primer BAss to beta actin [21]. A negative control reaction, with the reaction mixture lacking reverse transcriptase, was conduced for each reverse transcription reaction.

The sex of 12.5 dpc embryos and placentas was determined using the primers UB1X and UB1Y [21], which in vole giving a PCR products exclusively with male genomic DNA, and the strand-specific RT–PCR, detecting *Xist* expression exclusively in females (Fig. S1).

### 5′ and 3′ RACE

The exon–intron structure and boundaries of *Tsix* were analyzed using SMART RACE kit (Clontech). The gene-specific primers for 5′ and 3′ RACE are listed in Table S2. The amplified cDNA was cloned in plasmid vectors, sequenced, and analyzed by Southern blot hybridization. Vole *Tsix* cDNA obtained from RACE experiments was registered in GENE BANK under accession numbers [GenBank: JF519007] (*M. аrvalis*), [GenBank:JF519006], [GenBank:JF519005] (*M. rossiaemeridionalis*).

### Northern blot hybridization

About 5 µg of poly(A)+RNA isolated using Oligotex-dT30 (Qiagen) from vole and rat 14 dpc male embryos were run on a 1% agarose gel containing formaldehyde and transferred onto GeneScreen membrane (PerkinElmer). Both vole and rat cDNA amplicons corresponding to the exon located after the major *Tsix* start site in mouse [27] were obtained. The primer pairs used were gTF TCCTTCTGGCCTCTTCCGTCA - SNTR CTCTCCCTGCGCTCCCTCACT for vole and Rnt5 TCTAATATGACATTGCCGATG - Rnt6 GGCTCGCTTTCCGGACTATC for rat. For probe labeling 50 ng of the amplicons were added in linear PCR with [α^32^P]dCTP and strand-specific primers (gTF or Rnt5) producing DNA strand antisence to *Tsix* transcript. Hybridization was performed in x5 SSC, 0,5% SDS at 65°C overnight. Washing was carried out according to the manufacturer's instructions.

### Allelic expression assay

Comparison of the *M. arvalis* and *M. rossiaemeridionalis* Xics detected interspecific single nucleotide polymorphisms (SNPs) in the DNA sequences. The PCR primers (Table S3) were designed to generate the products containing SNPs. RT–PCR and PCR of genomic DNA were performed by standard methods using RNA and DNA samples of 12.5 dpc placentas of interspecific vole hybrids obtained from the crosses *M. rossiaemeridionalis* ×*M. arvalis*. As voles have imprinted XCI in extraembryonic tissues [26], the majority of placental cells have the inactive X chromosome of paternal origin and the active X chromosome of maternal origin. The PCR products containing SNPs were purified by extraction from agarose gel, and their nucleotide sequences were analyzed in an ABI Prism automated sequencer.

### DNA fluorescent in situ hybridizations

DNA FISH was carried out as previously described [54], [55]. The phage clones containing the sequences of the *M. rossiaemeridionalis* genes *Xist*, *Slc7a3*, *Chic1, Cdx4, Slc16a2,* and *Pgk1* labeled with biotin and digoxygenin were used as probes.

## Supporting Information

Figure S1 **Figure illustrating sexing of vole 12.5 dpc placentas and embryos.** (A) The sex of 12.5 dpc embryos and placentas was determined using the primers UB1X and UB1Y [21], which produce a PCR product exclusively from vole male genomic DNA. (B) Additionally, the strand-specific RT–PCR, which detects *Xist* expression exclusively in females, was performed. Strand-specific primer for *Xist* cDNA synthesis was SDX3, CCCAGTGCTGGTGAGCTATTCC. Subsequent PCR was performed with primers NSX19, GTGATTAATTCATTCTATCTGCC and MSX27, TTGCTCAGATTAGCTAG.
(TIF)

Figure S2 **Results of comparison of 3′ to** ***Xist*** **region in mouse and rat by PIP maker software.**
(PDF)

Figure S3 **Results of comparison of the vole sequence 3′ to** ***Xist*** **with the corresponding mouse region and** ***Slc7a3*** **by PIP maker software.**
(PDF)

Figure S4 **Figure illustrating localization of** ***Slc16a2*** **,** ***Cdx4*** **,** ***Chic1*** **,** ***Xist*** **and** ***Slc7a3*** **on the** ***M. rossiaemeridionalis*** **X chromosome.**
(TIF)

Figure S5 **Figure illustrating transcription antisence to** ***Xist*** **in rat.**
(EPS)

Table S1 **List of strand-specific primers for cDNA syntheses and PCR.**
(DOC)

Table S2 **List of gene-specific primers for 5′ and 3′ RACE.**
(DOC)

Table S3 **List of PCR primers used for allelic expression assay.**
(DOC)
