# Supplementary figures and images for: Variability of Sequence Surrounding the Xist Gene in Rodents Suggests Taxon-Specific Regulation of X Chromosome Inactivation
Source: PLoS One. 2011 Aug 3;6(8):e22771. doi: 10.1371/journal.pone.0022771 (PMC3149622; doi:10.1371/journal.pone.0022771)

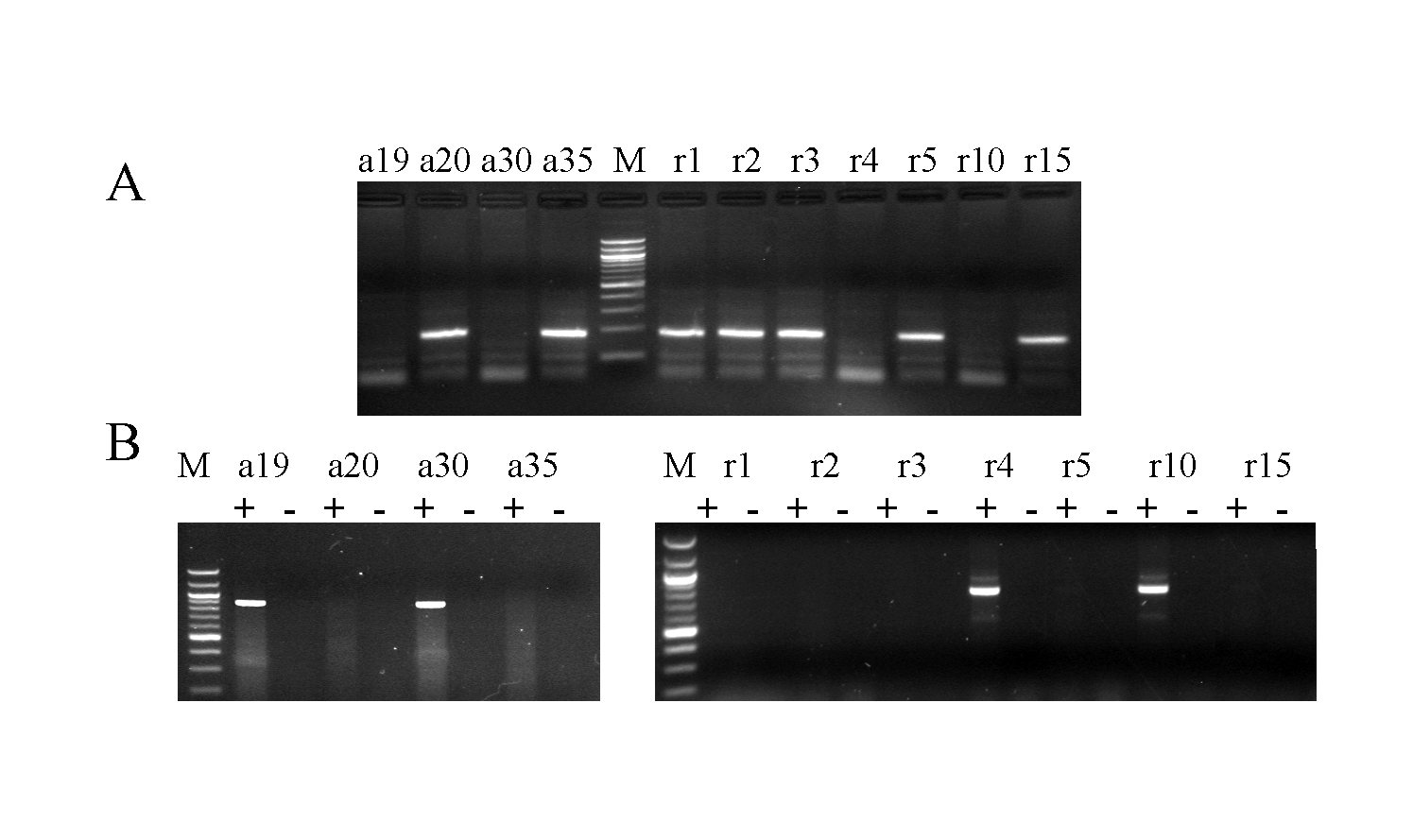

Supplement: Figure S1 — Figure illustrating sexing of vole 12.5 dpc placentas and embryos. (A) The sex of 12.5 dpc embryos and placentas was determined using the primers UB1X and UB1Y [21], which produce a PCR product exclusively from vole male genomic DNA. (B) Additionally, the strand-specific RT–PCR, which detects Xist expression exclusively in females, was performed. Strand-specific primer for Xist cDNA synthesis was SDX3, CCCAGTGCTGGTGAGCTATTCC. Subsequent PCR was performed with primers NSX19, GTGATTAATTCATTCTATCTGCC and MSX27, TTGCTCAGATTAGCTAG. (TIF) [file pone.0022771.s001.tif]

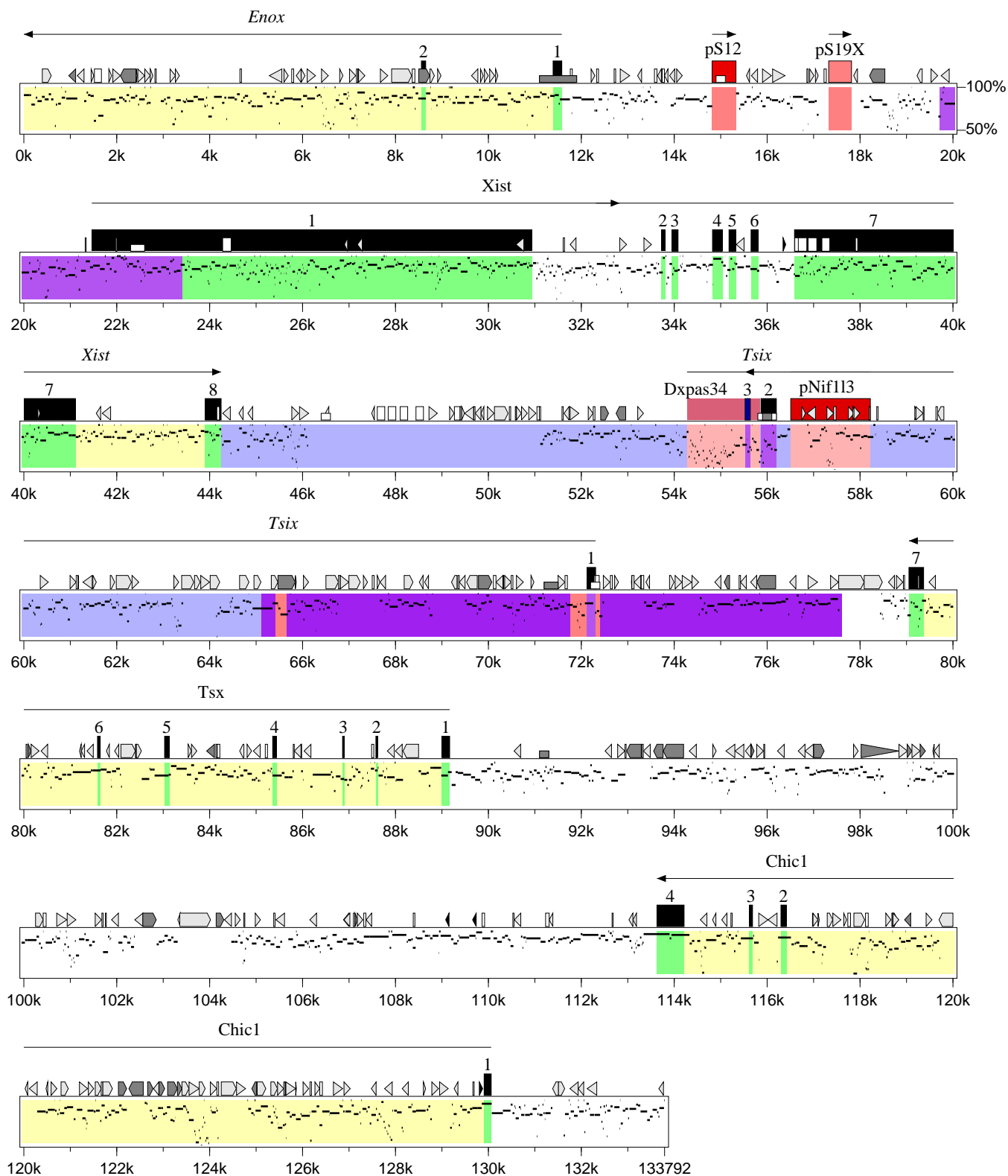

Supplement: Figure S2 — Results of comparison of 3′ to Xist region in mouse and rat by PIP maker software. (PDF) [file pone.0022771.s002.pdf]

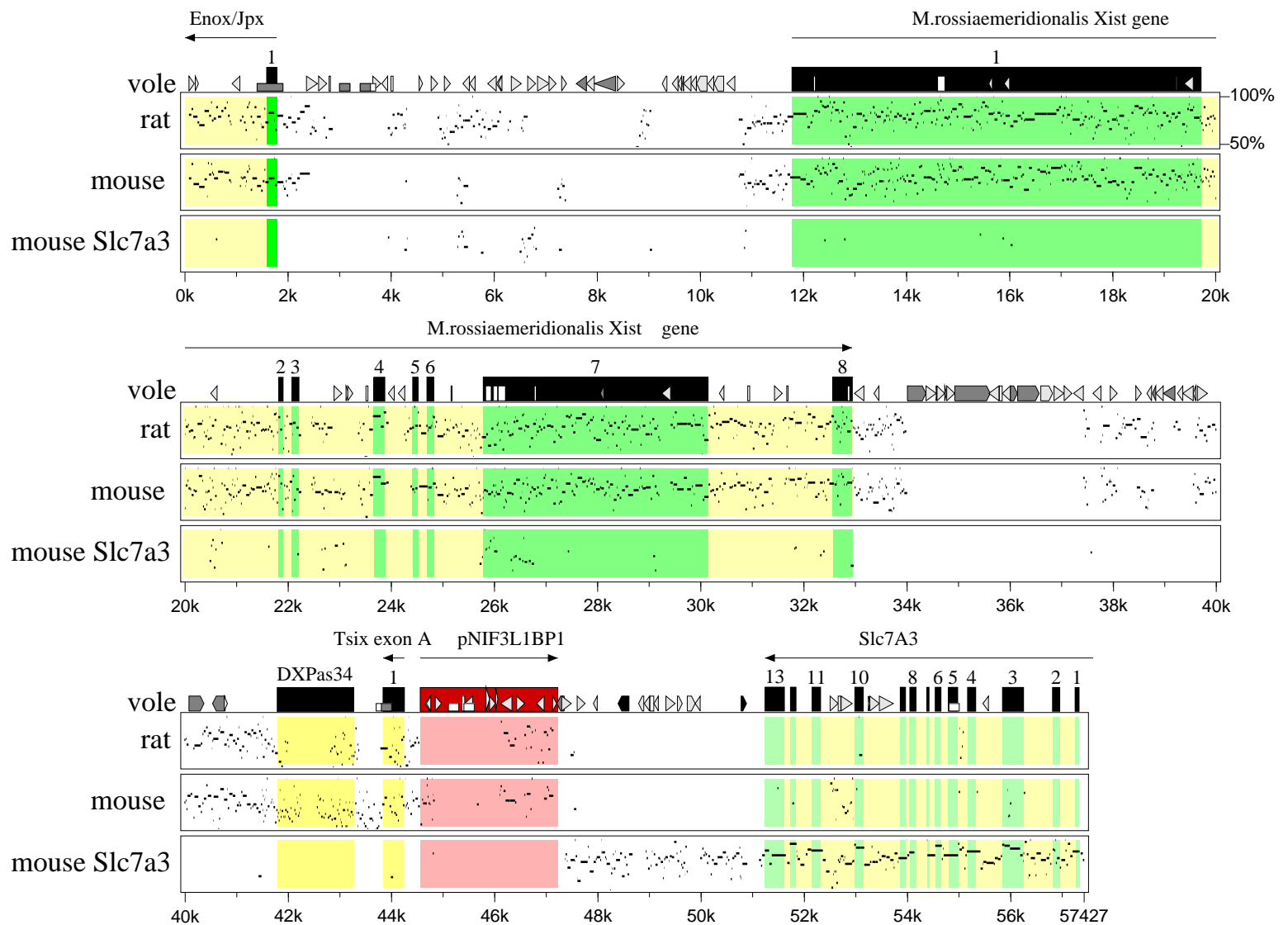

Supplement: Figure S3 — Results of comparison of the vole sequence 3′ to Xist with the corresponding mouse region and Slc7a3 by PIP maker software. (PDF) [file pone.0022771.s003.pdf]

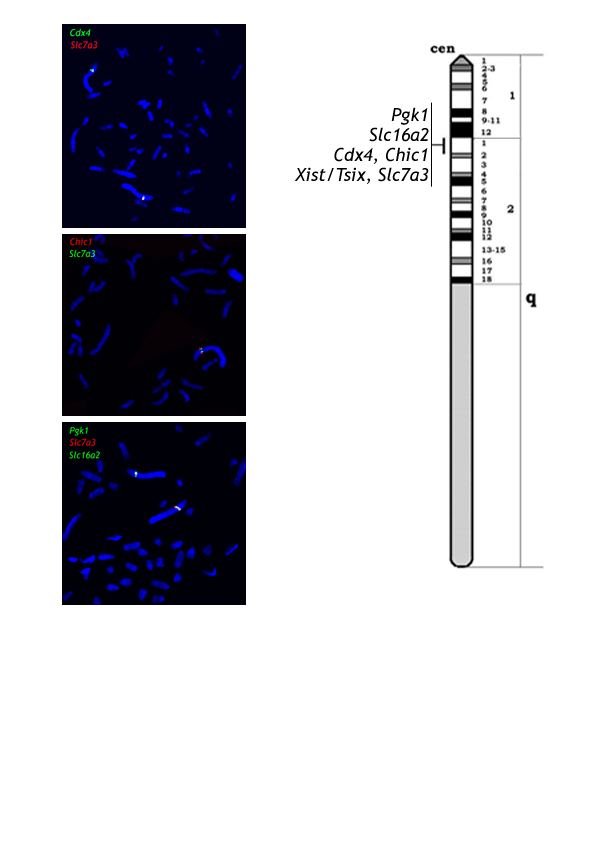

Supplement: Figure S4 — Figure illustrating localization of Slc16a2 , Cdx4 , Chic1 , Xist and Slc7a3 on the M. rossiaemeridionalis X chromosome. (TIF) [file pone.0022771.s004.tif]
