# Supplementary material for: Variability of Sequence Surrounding the Xist Gene in Rodents Suggests Taxon-Specific Regulation of X Chromosome Inactivation
Source: PLoS One. 2011 Aug 3;6(8):e22771. doi: 10.1371/journal.pone.0022771 (PMC3149622; doi:10.1371/journal.pone.0022771)
Supplement: Table S1 — List of strand-specific primers for cDNA syntheses and PCR. (DOC) [file pone.0022771.s006.doc]

Table S1. Strand-specific primers for cDNA syntheses and PCR

| Amplicon | Oligonucleotide sequences | Strand |
| --- | --- | --- |
| 1 | TCTATAACCAATACTCAGGACG  CTAATTTTTTCCCATGTATGTATC | AS  S |
| 2 | GACACATTAACTGGCCACTTTCTTCACTC  CCCTTCCGCCCCCGTTTTCTCAG | AS  S |
| 3 | ACAGACGTTAGCTGGAGGAAGTACG  TCCCTTTCTAATTTGTGTAGTAACCAG | AS  S |
| 4 | GACCTTCAATGTCCACTTACAG  TCCTCCAAGCCTGTCTCC | AS  S |
| 5 | CGCCTACTAAATGTGACTGTTCC  TTTCCTGCTTCCTTCATTGC | AS  S |
| 6 | TTGATTCTGTGAAGGGTGAGATAGG  GGACTTCCAAAGTTATGAGTCAACA | AS  S |
| 7 | CAATTTTGTGTTGTTATTCATCAG  GGTGCTTCACAAAATCGTTCCTT | AS  S |
| 8 | CCAACTGCCAGAAAAGTTATTG  AACAGTTACAGTGGTTTCTCCACA | AS  S |
| 9 | TAAAACCCCAAGACTAAGGGAAAT  AGACTAAAGATCAACCCCTGCAC | AS  S |
| 10 | TCCTTCTGGCCTCTTCCGTCA  CTCTCCCTGCGCTCCCTCACT | AS  S |
| 11 | GCCCATGTAGTAGACCGTTGTAT  GTGACTACTGTTTTTCTTGTGC | AS  S |
| 12 | CCAACAAAACAGGAGGCAAC  ACATTTCCGTGACTGGGAAG | AS  S |
| 13 | TGTTCTATGGCAAGCAGTTCC  ACAATGTTCTCTGGGGTTGG | AS  S |
| 14 | ATTCATGGCCTTCCTCTTC  TGAGAATGAGAACACAGATGG | AS  S |

Strand-specific primers for betta actin,

BAss, ACACGCAGCTCATTGTAG

PCR primers for betta-actin,

BA11, GATATCGCTGCGCTGGTCGT ,

BA2, AGATCTTCTCCATGTCGTCC

Note. AS, antisense; S, sense
