# Supplementary material for: Variability of Sequence Surrounding the Xist Gene in Rodents Suggests Taxon-Specific Regulation of X Chromosome Inactivation
Source: PLoS One. 2011 Aug 3;6(8):e22771. doi: 10.1371/journal.pone.0022771 (PMC3149622; doi:10.1371/journal.pone.0022771)
Supplement: Table S2 — List of gene-specific primers for 5′ and 3′ RACE. (DOC) [file pone.0022771.s007.doc]

Table S2. Gene-specific primers for 5’ and 3’ RACE

| **5’ RACE *Tsix***   | Name | Sequences | Comments | | --- | --- | --- | | MAX49 | GTGTGTGGGTTTGGACTTGAT | gene-specific primer for cDNA synthesis | | TSX6 | CTCTGTAGCTTTGGTG | gene-specific primer for PCR | |
| --- | --- | --- | --- | --- | --- | --- | --- | --- | --- |
| **3’ RACE *Tsix***   | SUPR6 | CCCGACTTCTTATTGGCGTTTTA | gene-specific primer located in *Xist* promoter, for PCR in *M. rossiaemeridionalis* | | --- | --- | --- | | T3aR | CGGATCAATTGGTGGGCTCGAGGTAAGGAA | gene-specific primer located in *Xist* promoter, for PCR in *M. arvalis* | | 3VR | CAAATCGGATGGTCATTCAACGCCTCAAAT | gene-specific primer located in the beginning of *Xist* exon 1 for PCR in both vole species | |
| **RT_PCR *Tsix***   | BT11 | TTCCTGCCCACCTTTATTAATGC | strand-specific primer for cDNA synthesis and PCR | | --- | --- | --- | | SNTR | CTCTCCCTGCGCTCCCTCACT | gene-specific primer for PCR | |
| **3’ RACE *Enox***   | SENOXR | CCCTTCCGCCCCCGTTTTCTCAG | gene-specific primer for PCR | | --- | --- | --- | |
| **5’ RACE *Anti-Enox***   | ANTIER | GCAATCCTGGCGGCTAAAACTACA | gene-specific primer for cDNA synthesis and PCR | | --- | --- | --- | |
