# Supplementary material for: Variability of Sequence Surrounding the Xist Gene in Rodents Suggests Taxon-Specific Regulation of X Chromosome Inactivation
Source: PLoS One. 2011 Aug 3;6(8):e22771. doi: 10.1371/journal.pone.0022771 (PMC3149622; doi:10.1371/journal.pone.0022771)
Supplement: Table S3 — List of PCR primers used for allelic expression assay. (DOC) [file pone.0022771.s008.doc]

Table S3. PCR primers used for allelic expression assay

| **Enox/Jpx**   | Name | Sequences | Comments | | --- | --- | --- | | SENOXF | GACACATTAACTGGCCACTTTCTTCACTC | The strand-specific primer for cDNA synthesis, PCR with cDNA, PCR with genomic DNA and sequencing | | SENOXR | CCCTTCCGCCCCCGTTTTCTCAG | The primer for PCR with cDNA, PCR with genomic DNA and sequencing | |
| --- | --- | --- | --- | --- | --- | --- | --- | --- | --- |
| ***Tsix***   | pXistF | CAATTTTGTGTTGTTATTCATCAG | The strand-specific primer for cDNA synthesis, PCR with cDNA, PCR with genomic DNA and sequencing | | --- | --- | --- | | pXist3VR | GGTGCTTCACAAAATCGTTCCTT | The primer for PCR with cDNA, PCR with genomic DNA and sequencing | |
| ***Xist***   | Name | Sequences | Comments | | --- | --- | --- | | SDX3 | CCCAGTGCTGGTGAGCTATTCC | The strand-specific primer for cDNA synthesis | | NSX19 | GTGATTAATTCATTCTATCTGCC | The primer for PCR with cDNA | | MSX27 | TTGCTCAGATTAGCTAG | The primer for PCR with cDNA | | Xist-SNP-F | CTTTTCTTCATGTGGCCTGCA | The primer for PCR with genomic DNA and sequencing | | Xist-SNP-R | AGGGATAATTCCGGCAGA | The primer for PCR with genomic DNA and sequencing | |
| ***Slc7a3***   | Slc8exF | ATTCATGGCCTTCCTCTTC | The strand-specific primer for cDNA synthesis, PCR with cDNA, PCR with genomic DNA and sequencing | | --- | --- | --- | | Slc8exR | TGAGAATGAGAACACAGATGG | The primer for PCR with cDNA, PCR with genomic DNA and sequencing | |
